# Safety and efficacy of temsirolimus as second line treatment for patients with recurrent bladder cancer

**DOI:** 10.1186/s12885-018-4059-5

**Published:** 2018-02-17

**Authors:** Marina Pulido, Guilhem Roubaud, Anne-Laure Cazeau, Hakim Mahammedi, Lionel Vedrine, Florence Joly, Loic Mourey, Christian Pfister, Alejandro Goberna, Barbara Lortal, Carine Bellera, Philippe Pourquier, Nadine Houédé

**Affiliations:** 1 Clinical and Epidemiology Department & Clinical Investigation Center INSERM CIC 1401, Bergonié Institute, Bordeaux, France; 2 Medical Oncology Department, Bergonié Institute, Bordeaux, France; 3 Nuclear Medicine Department, Bergonié Institute, Bordeaux, France; 4 Medical Oncology Department, Jean Perrin Cancer Center, Clermont-Ferrand, France; 5 Hartmann Oncology Radiotherapy Group, Levallois-Peret, France; 6 Medical Oncology Department, François Baclesse Cancer Center, Caen, France; 7 Medical Oncology Department, IUCT Oncopole, Toulouse, France; 8 Urology Department, Rouen University Hospital & Clinical Investigation Center INSERM CIC 1404, Rouen, France; 9 INSERM U1194, Montpellier Cancer Research Institute, Montpellier, France; 10 Medical Oncology Department, Nimes University Hospital, Nimes, France

**Keywords:** Metastatic bladder cancer, Clinical trial, Temsirolimus, mTOR

## Abstract

**Background:**

Bladder cancer is the 7th cause of death from cancer in men and 10th in women. Metastatic patients have a poor prognosis with a median overall survival of 14 months. Until recently, vinflunine was the only second-line chemotherapy available for patients who relapse. Deregulation of the PI3K/AKT/mTOR pathway was observed in more than 40% of bladder tumors and suggested the use of mTOR as a target for the treatment of urothelial cancers.

**Methods:**

This trial assessed the efficacy of temsirolimus in a homogenous cohort of patients with recurrent or metastatic bladder cancer following first-line chemotherapy. Efficacy was measured in terms of non-progression at two months according to the RECIST v1.1 criteria. Based on a two-stage optimal Simon’s design, 15 non-progressions out of 51 evaluable patients were required to claim efficacy. Patients were treated at a weekly dose of 25 mg IV until progression, unacceptable toxicities or withdrawal.

**Results:**

Among the 54 patients enrolled in the study between November 2009 and July 2014, 45 were assessable for the primary efficacy endpoint. A total of 22 (48.9%) non-progressions were observed at 2 months with 3 partial responses and 19 stable diseases. Remarkably, 4 patients were treated for more than 30 weeks. Fifty patients experienced at least a related grade1/2 (94%) and twenty-eight patients (52.8%) a related grade 3/4 adverse event. Eleven patients had to stop treatment for toxicity. This led to recruitment being halted by an independent data monitoring committee with regard to the risk-benefit balance and the fact that the primary objective was already met.

**Conclusions:**

While the positivity of this trial indicates a potential benefit of temsirolimus for a subset of bladder cancer patients who are refractory to first line platinum-based chemotherapy, the risk of adverse events associated with the use of this mTOR inhibitor would need to be considered when such an option is envisaged in this frail population of patients. It also remains to identify patients who will benefit the most from this targeted therapy.

**Trial registration:**

ClinicalTrials.gov Identifier: NCT01827943 (trial registration date: October 29, 2012); Retrospectively registered.

**Electronic supplementary material:**

The online version of this article (10.1186/s12885-018-4059-5) contains supplementary material, which is available to authorized users.

## Background

Bladder cancer is the seventh most common cancer worldwide in men and occurs at a median age of 73 years old [1]. Age-standardized incidence rates are higher in men (9 per 1000.000) than in women (2.2 per 100.000), which parallel the mortality rates of 3.2 and 0.9, respectively [2]. More than half of cases are occurring in the most developed areas including Europe and North America, but significant variations can be observed depending on the countries [2]. Though several risk factors have been invoked, it is admitted that tobacco use is the most prevalent one and could be associated with a future rise in incidence [3]. This represents a serious healthcare burden as bladder cancer is associated with one of the highest treatment costs [4]. Most bladder cancers are urothelial carcinomas and include the two categories of non-muscle-invasive and muscle-invasive tumors, the latter representing 20–30% of newly diagnosed cases [5]. While non-muscle invasive tumors are usually of good prognosis, up to 25% of them progress to the invasive form of the disease [6]. Transurethral resection of the bladder is the treatment of choice for non-muscle-invasive bladder cancers and cystectomy is used for non-metastatic forms of muscle invasive tumors [7]. In the case of locally advanced tumors or in metastatic diseases, two first-line chemotherapies where cisplatin is associated with either gemcitabine (GC) or methotrexate, vinblastine, and doxorubicin (MVAC) have been approved and show overall response rates above 50% with a median progression-free survival (PFS) of 7–9 months and a median overall survival (OS) of 12–15 months [8]. Vinflunine was the only drug approved in 2009 as second line therapy based on a 2.4 months benefit as compared to best supportive care [9], emphasizing the need for new treatment options. For these patients, blockade of the PD1/PD-L1 immune checkpoint is an attractive strategy as recent phase II/III clinical trials showed significant improvement in tumor response, with a higher response rate for patients with PD-L1 positive tumor-infiltrating immune cells and a good tolerability [10]. This led to the approval of pembrolizumab, atezolizumab, durvalumab, nivolumab and avelumab as second line treatment for platinum pretreated patients [11–15].

With the implementation of tumor collections and the development of new generation sequencing, a growing number of potential actionable mutations have been identified in solid tumors. In bladder cancers, numerous gene alterations have been reported in a fare percentage of tumor samples including PTEN deletions, mutations of FGFR3, TP53, RAS or RAF or mutations of several key factors of the PI3K/Akt/mTOR signalling pathway (reviewed in [16]). Deregulation of the PI3K/Akt/mTOR pathway was associated with remarkable efficacy of mTOR inhibitors such as rapamycin or everolimus in bladder cancer cells in vitro and in xenograft models [17–20]. Temsirolimus or everolimus are approved for the treatment of metastatic renal cell carcinoma, breast cancer, mantle cell lymphomas and neuroendocrine pancreatic tumors [21–25].

Two phase II trials have been conducted with everolimus in patients with urothelial cancer who were refractory to first line platinum-based chemotherapy and showed mild antitumor activity [26, 27]. The study of Seront et al. reported a prevalence of PTEN loss in non-responder patients,^21^ and only few prolonged responses were associated with the presence of specific mutations of the TSC1 gene [28]. A phase II trial was also conducted with temsirolimus in the same settings, but was stopped after the inclusion of 14 patients as no sufficient benefit was observed on OS, leading to the conclusion that temsirolimus had poor activity [29].

Here, we report the results of a multicenter, open-label, single-arm phase II trial evaluating the antitumor activity of temsirolimus in patients with relapsed bladder cancer after first-line chemotherapy. The primary objective was to evaluate the efficacy of temsirolimus in terms of two months non-progression. Secondary objectives were to evaluate PFS and OS as well as toxicity. Our study is the first to provide clinical evidence of a potential benefit of temsirolimus for the treatment of relapsed bladder cancers. We also discuss the potential use of PET scan (early-D15 and late-D56 diagnostic performances) as a predictive value of response to temsirolimus.

## Methods

### Study design

This is a French multicentre, two-stage, phase II, single-arm, open label clinical trial based on Simon’s two-stage design assessing the efficacy and safety of temsirolimus (Torisel®) in patients with urothelial carcinoma of the bladder who relapse after first-line chemotherapy. This study is registered at ClinicalTrials.gov, number NCT01827943 [30]. It was approved by each local ethics committee and by the regulatory agencies and was conducted according to the good clinical practices and the declaration of Helsinki.

### Patient selection

Inclusions started in November 2009 from six University Hospitals and Clinics in France.

Eligible patients had to be 18 years or older with histologically proven locally advanced or metastatic (stage IV) bladder cancer, an ECOG status ≤2 and a documented relapse following treatment with a first line platinum-based chemotherapy. All patients included in this trial had received only a single line of chemotherapy for metastatic disease prior to temsirolimus. Patients had measurable lesions on at least one dimension on CT scan according to RECIST criteria v1.1 and did not receive antineoplastic therapy 4 weeks before inclusion. Also, blood tests were compatible with temsirolimus prescription following prescription rules indicated for kidney cancer, most importantly neutrophils > 1500, platelets > 100,000 and creatinine clearance > 40 mL/min. Patients with brain metastases (whether symptomatic or not), hypersensitivity to temsirolimus and who received chemotherapy within a 4 weeks period prior to inclusion were excluded from the study. All patients provided a written informed consent prior to inclusion.

### Treatment and outcomes

Temsirolimus was administered intravenously at a dose of 25 mg in a weekly 30 min infusion and was associated to anti-H1 treatment. One cycle corresponded to 4 weeks of treatment. Temsirolimus efficacy was evaluated in terms of non-progression rate at eight weeks. According to RECIST criteria version 1.1 [31], non-progression is defined as complete (CR) or partial response (PR) or stable disease (SD). All partial or complete responses were confirmed four weeks after the initial documentation by a central blinded radiology review of all imaging.

Secondary outcomes included duration of overall response, one-year progression-free survival (PFS), one-year overall survival (OS) and toxicity. PFS is defined as the time from the initiation of treatment to the time of progression, according to RECIST 1.1 criteria, or death from any cause. OS is defined as the time from the initiation of treatment to death from any cause. Duration of overall response is defined as the time from the first radiological examination showing partial or complete response (whichever is first recorded) to the objectively documented progression of disease. To be assessable for the primary endpoint, patients had to meet eligibility criteria and received at least one dose of treatment.

### Toxicity

Toxicity analysis was performed on all patients who received at least one administration of the study drug. Tolerability and safety were assessed through recording of adverse events using Common Terminology Criteria for Adverse Events from NCI (Version 3.0).

### FDG-pet/ct

Optional study included PET-scan analysis that was performed at the time of inclusion visit and 15 and 56 days after inclusion to evaluate early and late diagnostic performance of PET-scan and concordance of PET-scan with CT-TAP Scan.

Three PET centres were involved using three different devices but a similar acquisition protocol. After 6 h of fasting, the blood glucose level of each patient was measured, and the patient was injected with 3 MBq/Kg of 18F FDG. One hour later, a CT scan without contrast agent was performed, covering the area from the vertex to the proximal thigh, and the images were used for attenuation correction and image fusion. This was followed by whole-body 3D PET acquisition with eight bed positions according to local procedure of emission scan time each using a dedicated PET/CT scanner.

### Image interpretation

FDG-PET/CT scans were centralized reviewed by an experienced blinded nuclear medicine physician. Baseline and subsequent PET were compared for each patient using the EORTC criteria. For each patient, a maximum of 5 lesions were selected at baseline with the highest 18F–FDG uptake in as many organs as possible and measured at the follow-up scans. We measured SUVmax according to body surface area as required in EORTC criteria (SUVmaxbsa) and SUVmax according to bodyweight (SUVmaxbw). SUVmaxbsa measurements from all target lesions were summed on each scan, giving ∑SUVmax. At the first follow-up and if ∑SUVmax was decreasing compared with baseline, response was calculated as Δ∑SUVmax between baseline and actual follow-up divided by baseline ∑SUVmax • 100%. If SUVmax increased, response was calculated as Δ∑SUVmax between lowest registered and actual follow-up divided by lowest registered ∑SUVmax • 100%. Response was classified according to the 4 EORTC categories: complete metabolic response (CMR) with complete resolution of 18F–FDG uptake within all lesions; partial metabolic response (PMR) with a reduction of ∑SUVmax of 15–25% after 1 cycle and at least 25% after more than 1 cycle; progressive metabolic disease (PMD) with a ≥ 25% increase of ∑SUVmax corresponding to a visible increase in 18F–FDG uptake (> 20% in the longest dimension) or to a new 18F–FDG–avid lesion; stable metabolic disease (SMD) with a response between PMR and PMD.

### Sample size

Sample size estimation was performed based on the Simon’s optimal two-stage design [32] and calculated with the npII software [33]. We used unacceptable and acceptable response rates of 20% and 40%, respectively. We believed that unacceptable response rate of 20% was clinically relevant for the development of such targeted therapies in such an indication and that 40% would correspond to the maximum effect that we could hopefully obtain. It is usually admitted that PFS is probably more relevant for the evaluation of a targeted therapy as compared to tumor shrinkage used for cytotoxic agents. Indeed mTOR inhibitors were developed based on the evaluation of PFS in renal cancer. We used respectively, a 5% type I error rate and a 10% type II error rate (85% power). The choice of 85% power was chosen deliberately in order to reduce the number of patients that would be necessary to evaluate temsirolimus efficacy in such a fragile patient population for whom very few alternatives could be offered at the time of the trial design. With an anticipated drop-out rate of 10%, 55 subjects were necessary. At the end of the first stage, the trial would be continued if a minimum of 5 non-progressions were observed among 17 assessable patients. At the second stage 34 additional assessable subjects would be recruited and ≥15 responses would be required to claim efficacy.

### Data analysis

All analyses were descriptive; no *p*-values were calculated. Categorical endpoints were reported in terms of counts and proportions. Non-progression rate at 8 weeks was estimated using binomial estimates and reported with its 95% confidence interval (CI). Continuous endpoints were reported in terms of summary statistics including number of patients, median, minimum, and maximum. Survival endpoints (PFS and OS) were analysed using the Kaplan-Meier method. The median survival rates were reported with a 95% CI. Median follow-up was calculated using the reverse Kaplan-Meier method.

## Results

### Patients and treatment

**Fig. 1:**
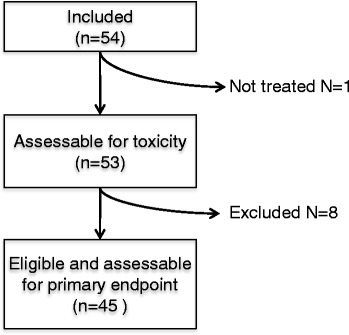
Flowchart of patient inclusions into the current study

This multicentre study was conducted in six centres between November 2009 and July 2014. Among the 54 patients who were enrolled, 9 did not meet the eligibility criteria. One patient was never treated because of a rapid progression of the disease. The 8 other patients had major protocol deviations: ECOG 3 status (1), upper tract urothelial carcinoma (1), prostate adenocarcinoma pT2 (1), two lines of chemotherapy for metastatic disease (2), non-measurable disease (1), unknown primary tumor (2). *In fine*, 45 patients were assessable for primary efficacy outcome (Fig. 1). Baseline demographic characteristics are summarised in Table 1. Median age was 65 years old (range 41–87) and the majority of included patients were men (42 men; 77.8% versus 12 women, 22.2%). Some patients received perioperative adjuvant (24%) and neoadjuvant chemotherapy (11%). Patients were treated for metastatic disease (81.5%) or locally advanced tumor (18.5%).

**Table 1:** Patient characteristics

Population (*N* = 54)		
	Median	[range]
Age	65	[41–87]
	N	%
Sex		
Men	42	77.8
Women	12	22.2
ECOG		
0	20	37.0
1	25	46.3
2	8	14.8
3	1	1.9
Stage of the tumor at diagnosis		
0	3	5.6
1	15	27.7
2	13	24.1
3	7	13.0
4	16	29.6
Primary Location		
Upper Urinary Tract	3	5.6
Bladder	51	94.4
Type of predominant cells		
transitional cell carcinoma	31	57.4
Other	14	25.9
Not available	9	16.7
Type of cancer at time of enrollment		
Metastatic	44	81.5
Locally advanced	10	18.5
Sites of metastasis (could be more than one)		
Lung	20	37.0
Pleura	1	1.9
Liver	21	38.9
Bones	22	40.7
Brain	2	3.7
Nodes	27	50
Other	11	20.4
Neoadjuvant Chemotherapy		
Yes	6	11.1
No	48	88.9
Adjuvant Chemotherapy		
Yes	13	24.1
No	41	75.9

### Outcomes

**Fig. 2:**
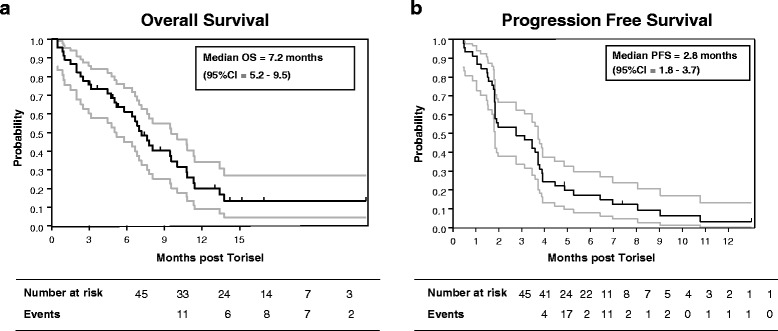
Kaplan-Meier curves illustrating overall survival (**a**) and progression-free survival (**b**) for all patients who were entered into this study

**Fig. 3:**
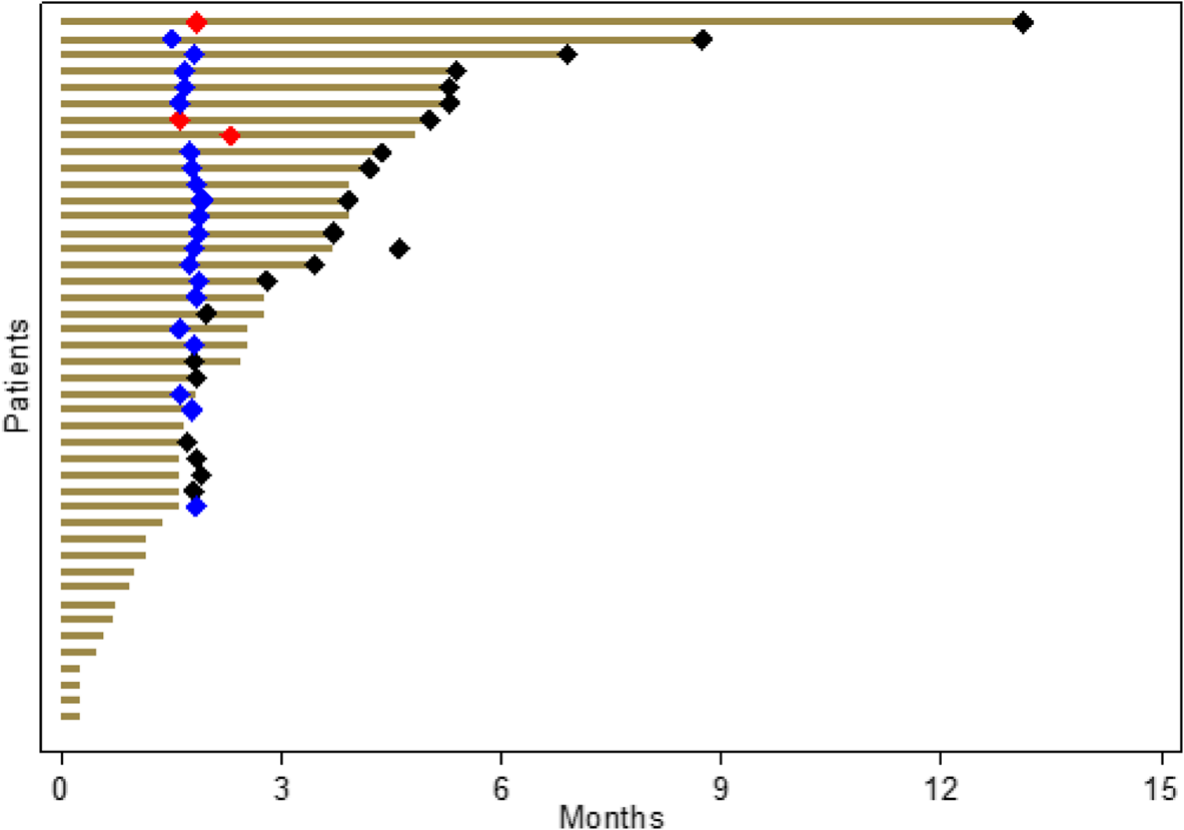
Swimmer plot indicating the number of patients treated with temsirolimus as a function of treatment duration. Red diamonds: PR; blue diamonds: SD; black diamonds: progression or death

Of the first 17 patients included for efficacy, 10 patients were assessable for the primary endpoint and 6 non-progression were observed. Even though at the time of the interim analysis the number of 17 assessable patients was not reached, the primary objective was met (> 5 non-progression) allowing the steering committee to continue to stage two. Then, 37 additional patients were included. At that stage, 45 patients were assessable for the primary endpoint. Three partial responses and 19 stable diseases were observed for a total of 22 non-progressions at eight weeks, leading to a non-progression rate of 48.9% (95% CI 33.7–64.2) (Table 2). According to RECIST criteria, 10 patients had progressive disease and 13 patients could not be evaluated because of death (12 patients) or to treatment discontinuation due to toxicity (one patient). Median overall survival (OS) was 7.2 months (95% CI = 5.2–9.5) and progression-free survival (PFS) was 2.8 months (95% CI = 1.8–3.7) (Fig. 2). Remarkably, 4 patients were treated for more than 30 weeks (Fig. 3).

**Table 2:** Patients’ responses evaluated at 8 weeks according to the RECIST criteria

Non-progression rate at 8 weeks (*N* = 45)		
Rate % (CI 95%)	48.9	(33.7–64.2)
Response at 8 weeks (*N* = 45)		
	N	%
Response at 8 weeks (*N* = 32)		
Complete response	0	0.0
Partial response	3	6.7
Stable disease	19	42.2
Progressive disease	10	22.2
Not evaluated 8 weeks (*N* = 13)

### Safety

Fifty-three patients received at least one IV of temsirolimus and were assessable for toxicity analyses. Most patients received one or two cycles of treatment (12 and 13, respectively), five patients received three cycles, three received four and six cycles, two patients received five, one patient seven cycles and two patients received up to 10 cycles. The median duration on temsirolimus treatment was 9 weeks. Twenty seven patients (50.9%) had at least one dose hold and 8 patients (15.1%) had dose reduction.

Treatment was tolerable and no unknown or unexpected adverse event (AE) was reported. Most commonly experienced toxicities all grades were gastrointestinal (73.6%) and constitutional symptoms including fatigue, fever, insomnia, sweating and weight loss (62.3%) (Table 3). The most common grade 3 or 4 AE were haematological toxicity (18.9%), constitutional symptoms (18.9%) and gastrointestinal toxicity (11.3%). In total fifty patients experienced at least a related grade1/2 (94%) and twenty-eight patients (52.8%) a related grade 3/4 adverse event. Eleven patients (20.8%) discontinued treatment because of grade 3 or 4 AE leading to recruitment being halted by an independent data monitoring committee with regard to the risk-benefit balance and because of a rather slow recruitment pace and the fact that the primary objective was already met.

**Table 3:** List of adverse events from Grade 1/2 and Grade 3/4 evaluated according to the NCI-CTCAE (Version 3.0) toxicity classification

System Organ Class	Adverse Event (CTCAE V3.0) - Number of pts. (%)			
	Grade			
	Grade 1/2		Grade 3/4	
	*N*	%	*N*	%
Blood/Bone Marrow	16	30.2	10	18.9
Gastrointestinal	39	73.6	6	11.3
Asthenia/Fatigue	33	62.3	10	18.9
Dermatology/Skin	23	43.4	2	3.8
Metabolic/Laboratory	19	35.8	4	7.5
Hyperglycemia	3	5.7	1	1.9
Hyperlipidemia	11	20.8	2	3.8
Infection	12	22.6	2	3.8
Neurology	7	13.2		
Pulmonary/Upper Respiratory	6	11.3		
Renal/Genitourinary	3	5.7	1	1.9
Cardiac General	1	1.9	2	3.8
Vascular			1	1.9

### Optional analysis

PET scans were performed at inclusion for 24 patients. Among these patients, 13 patients had a PET scan at day 15 and 8 patients at day 15 and day 56 and were assessable for early and late diagnostic performance (Additional file 1: Table S1). Partial metabolic response was observed in 10 patients with PET scan at baseline and day 15 and in 5 patients with PET scan at baseline, day 15 and 56. These results indicate that early PET scan evaluation (day 15) could only be predictive of temsirolimus response for 6 patients out of 13 (46%).

## Discussion

Management of patients with metastatic urothelial carcinoma of the bladder following treatment with platinum-based chemotherapy remains a major challenge given the difficulty to control these symptomatic patients and avoid severe adverse events. Prior the immunotherapy era, potential options in the therapeutic armamentarium included kinase inhibitors targeting the PI3K/AKT/mTOR pathway that was known to be activated in bladder cancer [16, 34]. Indeed, preclinical studies using bladder cancer cell models or xenografted mice demonstrated that inhibition of mTOR by everolimus inhibited bladder cancer cell growth in vitro and in xenografted mice [19]. Subsequently, clinical trials evaluating the mTOR inhibitors everolimus or temsirolimus in this population of patients have been performed but failed to meet their primary objectives despite some prolonged responses [26, 27, 29].

The results of our multicenter single-arm phase II trial showed for the first time a clinical benefit of temsirolimus for the treatment of patients with relapsed bladder cancer after first-line chemotherapy. In terms of efficacy, our study showed a two-month non progression rate of approximately 50% and an overall survival that reached approximately 7 months (5.2–9.5 CI95%). This result differs from the poor activity of the sole other trial evaluating temsirolimus as a single agent in that indication, with a median time to progression of 2.5 months and an overall survival of 3.5 months [29]. This could easily be explained by the small number of patients who were assessed (14 patients) due to the premature closing of the trial because the endpoint was not met, but it could also be attributed to a higher proportion of patients with advanced disease and poor prognosis [29]. The objective response rate of 6.7% that we observed for temsirolimus was however lower than response rates obtained in phase II/III trials testing tubulin poisons in second line setting. In the SECAVIN trial comparing cabazitaxel and vinflunine, objective response rates were of 13% and 30%, respectively [35]. In the recent KEYNOTE-045 trial comparing chemotherapy to the immune checkpoint inhibitor pembrolizumab, a response rate of 11.4% was observed in the chemotherapy arm (paclitaxel, docetaxel or vinflunine) [12]. In terms of toxicity, both trials showed a similar safety profile with equivalent rates (20%) of grade 3–4 adverse events (mainly hematologic toxicity and fatigue), indicating an acceptable tolerance in patients with an ECOG status ≤2 and a rate of visceral metastases of approximately 80%, which is highly representative of such kind of population.

The first phase II trial with everolimus reported a clinical activity with a 2-month non progression rate of 27% in a cohort of 28 assessable patients [26]. The second study was performed in a larger cohort of 45 patients and reported a 2-month non-progression rate of 51%, which is almost identical to the results of our study, despite the enrollment of a significant proportion of patients who received 2 or more additional lines of treatment following first line platinum-based chemotherapy [27].

More recently, a dual inhibitor targeting PI3K and mTORC1/2 complexes, BEZ235 was tested in urothelial carcinoma after failure of platinum-based chemotherapy [36]. Notwithstanding an unfavorable toxicity profile, few patients experienced a clinical benefit [36]. Other trials investigating the PI3K inhibitor BKM-120 or the mTORC1/2 inhibitor AZD8055 are currently ongoing and results are pending.

Together with previous studies, the results of our clinical trial reinforce the notion that blockade of the PI3K/AKT/mTOR pathway could be beneficial for a specific subset of platinum-refractory patients. However, identification of robust predictive marker of response to mTOR inhibitors is still needed to select potential responder patients. In the case of everolimus, PTEN loss was associated with poor response to the drug, whereas responder patients showed a reduction in the expression levels of proteins involved in angiogenesis [26]. Interestingly, everolimus induced some spectacular response with treatment duration of more than 26 months for one patient [27]. It was further shown that this patient harbored mutations of negative regulator of mTOR such as TSC1 that could result in a significant change in response duration as demonstrated further in other patients [37]. Similar to that study we identified 4 patients experiencing a durable response to temsirolimus of more than 30 weeks (31, 33, 43 and 52 weeks, respectively). It is possible that these patients could also harbor TSC1 mutations or other alterations of the PI3K/AKT/mTOR pathway. Though it is feasible, measuring the activity of the PI3K/AKT/mTOR pathway would need to be performed in metastases to ensure a better predictive value [36]. In the present study, we also evaluated the potential predictive value of early PET scan in the tumor response to temsirolimus, as it was suggested to be a more accurate diagnostic tool as compared to CT scan [38]. We found that early PET scan (performed at 15 days) could predict response to temsirolimus at 2.8 months in less than half of treated patients, indicating that it cannot be used as a reliable marker.

When our study was started, no result of trials investigating immune checkpoint inhibitors targeting PD1 or PD-L1 was available. However, these trials led to the approval of 5 immune checkpoint inhibitors (pembrolizumab, atezolizumab, durvalumab, nivolumab and avelumab) in platinum pretreated patients based on durable response with objective response rates varying from 16 to 21% and overall survival ranging from 8 to 13 months and a superiority to chemotherapies [11–15]. These studies also revealed that response to anti-PD-L1 inhibitors was associated with increased levels of PD-L1 expression on immune cells and relied on mutation load within the tumors [11], which probably explains why these immunotherapies are actually efficient in only 25% of this specific population of patients (Reviewed in [39]). In that context mTOR inhibitors should be considered as a relevant option for patients who are not responding to immune checkpoint inhibitors and could either be used after relapse or in combination as it is currently being tested [40]. It is difficult to predict what the response rate would be for immune checkpoint-pretreated patients, a situation that could not be envisaged at the time of our study design, nor it is possible to predict response when immune checkpoints inhibitors would be combined to mTOR inhibitors. However, clinical trials testing these strategies will probably provide us with these results in the near future.

## Conclusion

Until recently there were only few options for patients with metastatic bladder cancers after failure to cisplatin-based chemotherapy. The recent approval of several immunotherapies really made a difference with durable responses that were not observed before and they will certainly occupy a major place in the armamentarium. Despite the positivity of this trial suggesting that termsirolimus might be a potential option for a subset of patients who are refractory to first line platinum-based chemotherapy, the risk of adverse events linked to the use of this mTOR inhibitor would have to be considered when such an option is envisaged for such a fragile population of patients, given that robust biomarkers could be identified to select the potential responders [41].

## Additional file

Additional file 1: Table S1. Metabolic response evaluated by PET-scan Description: Global metabolic response evaluated by PET-scan at day 15, or at day 15 and 56 as compared to baseline and patients’ status at 2.8 months. R: Responder; NR: Non Responder. (DOCX 25 kb)

## Abbreviations

mTOR: mammalian Target of Rapamycin
OS: Overall survival
PFS: Progression-free survival
