# Supplementary material for: Safety and efficacy of temsirolimus as second line treatment for patients with recurrent bladder cancer
Source: BMC Cancer. 2018 Feb 17;18:194. doi: 10.1186/s12885-018-4059-5 (PMC5816357; doi:10.1186/s12885-018-4059-5)
Supplement: Supplementary file 1 — Metabolic response evaluated by PET-scan Description: Global metabolic response evaluated by PET-scan at day 15, or at day 15 and 56 as compared to baseline and patients’ status at 2.8 months. R: Responder; NR: Non Responder. (DOCX 25 kb) [file 12885_2018_4059_MOESM1_ESM.docx]

**Supplementary Material**

**Supplemental Table 1**: Global metabolic response evaluated by PET-scan at day 15, or at day 15 and 56 as compared to baseline and patients’ status at 2.8 months. R: Responder; NR: Non Responder
